# Learning from Less: Pose-Balanced Sampling for High-Performance Face Recognition

**DOI:** 10.3390/s26216937

**Published:** 2026-11-02

**Authors:** Jhon I. Pilataxi, Jorge E. Zambrano, Robert Guamán-Rivera, Rodrigo Olivares

**Affiliations:** 1 Escuela de Ingeniería Informática, Universidad de Valparaíso, Valparaíso 2362905, Chile; rodrigo.olivares@uv.cl; 2 Escuela de Informática y Telecomunicaciones, Universidad Diego Portales, Santiago 8370190, Chile; jorge.zambrano@mail.udp.cl; 3 Departamento de Ingeniería de Sistemas y Computación, Universidad Católica del Norte, Antofagasta 1270709, Chile; robert.guaman@ucn.cl

**Keywords:** data-efficient training, face recognition, frontal–profile verification, pose-balanced sampling, pose variation

## Abstract

Large-scale face recognition (FR) datasets are strongly skewed towards frontal images, which limits the ability of models to learn pose-robust representations and leaves frontal-profile verification as a persistent source of error. This paper proposes a pose-balanced sampling strategy that builds a compact training subset by conditioning selection on the per-identity head-pose distribution rather than on sample difficulty or redundancy. Yaw angles are estimated with 6DRepNet, and an equal number of frontal and profile images are retained for every identity. Applied to MS1Mv3, the strategy yields MS1Mv3-FP, a subset of 547,202 images (10.7% of the original) covering 78.5% of the identities. An SE-ResNet100 trained from scratch on MS1Mv3-FP using the ArcFace loss reaches up to 98.73%, 93.23%, and 94.74% verification accuracy on CFP-FP, CPLFW, and VGG2-FP (mean over three sampled subsets: 98.63%, 93.13%, and 94.50%), within 0.30, 0.70, and 0.86 percentage points (pp) of the same network trained on the complete dataset, while reducing training time from more than 100 h to approximately 11 h on a single GPU. Equal-size subsets built without pose balancing lose 0.8–3.6 pp on average, indicating that the pose composition of the subset, and not only its size, accounts for this result. Frontal–frontal verification accuracy is largely preserved, whereas age-gap verification degrades, delimiting the scope of the strategy. The resulting reduction in training time and data could support the development of pose-robust FR models for camera-based sensing systems operating under resource constraints.

## 1. Introduction

The digitization of information has driven the development of systems that automatically process and analyze visual data, with face recognition (FR) among the most widely used biometric technologies in surveillance, access control, authentication, and human–machine interaction [1,2,3,4]. FR comprises two main tasks: verification (1:1), which determines whether two images correspond to the same person [5], and identification (1:N), which determines a person’s identity by matching the image against a gallery [2].

In camera-based sensing systems, images are typically acquired with individuals at varying positions and orientations relative to the imaging device. Unconstrained acquisition conditions can produce substantial variation in the observed head pose [6,7]. Such pose variations directly affect the information available to downstream recognition algorithms and can reduce verification reliability when frontal or near-frontal samples dominate the training data. Therefore, the head-pose distribution in FR datasets matters not only for recognition performance but also for the design of robust camera-based sensing systems.

Earlier FR systems relied on handcrafted representations, such as statistical local descriptors and their ensembles [8,9], whose discriminative power degrades under large pose variations. Deep-learning-based methods have become central to FR system development because they can learn discriminative facial representations from large-scale datasets [4,10]. Under unconstrained conditions, the performance of deep-learning-based FR systems depends primarily on model design and training, with three factors playing a particularly important role: network architecture, loss function, and training data [4,11].

The evolution of FR systems has closely followed the development of increasingly deep and discriminative architectures [12]. Models originally proposed for image classification, such as VGGNet, GoogLeNet, ResNet, and SENet, have been adapted to extract facial representations [4]. In particular, ResNet-based architectures such as ResNet50 and ResNet100 have become standard backbones for developing and evaluating FR models [1,4,13].

Progress has also been driven by training objectives: margin-based loss functions such as CosFace [14], ArcFace [15], CurricularFace [16], AdaFace [17], and UnifiedFace [18] have improved the discrimination between identities. More recent methods have further refined the learned embedding space, for example, through topology alignment (TopoFR) [19] or an intra-class incoherence constraint (IIC) [20]. Furthermore, large-scale datasets such as CASIA-WebFace [21], VGGFace2 [22], MegaFace [23], and MS1M and its refined versions, such as MS1Mv3, have expanded the volume and diversity of data available for training FR models [24,25]. Studies that introduce new loss functions or larger training datasets also tend to retain ResNet50 and ResNet100 as baseline architectures [1,4,26].

Despite these advances, pose variation remains one of the most persistent challenges in unconstrained FR [27,28]. State-of-the-art models routinely surpass 99.8% verification accuracy on the Labeled Faces in the Wild (LFW) dataset [15], which mostly contains near-frontal faces. Nevertheless, their performance drops by several percentage points on cross-pose benchmarks such as Cross-Pose LFW (CPLFW) [29] and the frontal–profile protocol of the Celebrities in Frontal-Profile dataset (CFP-FP) [30], where frontal images are compared against profile images. Several strategies have been proposed to narrow this frontal–profile gap, including pose-invariant loss designs such as FCGFace [31], feature-space frontalization approaches [32], and neuroevolutionary designs for pose-specialized architectures [27]. However, these solutions typically introduce additional architectural complexity, auxiliary modules, or costly architecture search procedures.

In addition, training high-capacity models on ever-larger datasets poses both computational and methodological challenges. Training or fine-tuning FR models with millions of images requires significant computational, energy, and storage resources, which may limit the reproducibility of experiments in resource-constrained environments [33,34]. Furthermore, increased data volume does not, by itself, guarantee a proportional improvement in generalization, particularly when the data exhibit non-uniform distributions.

Large-scale FR datasets show a marked imbalance in pose distribution, with far more frontal and near-frontal images than profile images [27,30]. In MS1Mv3, for example, frontal images outnumber profile images by about 9:1 (2,585,593 frontal vs. 279,837 profile images). This distribution may limit the ability of models to learn pose-robust representations, even as the total number of training images increases. Recent work has shown that up to 50% of the training data can be removed without a significant loss in accuracy [34]. However, existing pruning methods select samples primarily based on prediction dynamics or redundancy criteria, without explicitly controlling the pose distribution of the resulting subset.

These findings raise the question of whether a smaller training subset with a more balanced pose distribution can maintain competitive face verification performance. To address this question, we explicitly balance frontal and profile images while substantially reducing the amount of training data. Specifically, we propose a pose-balanced sampling strategy that reduces redundancy in the training data while preserving a balanced representation of poses for each identity. Images of each identity are classified as frontal or profile according to the yaw angle estimated by 6DRepNet [35]. Applying this strategy to MS1Mv3 produces MS1Mv3-FP, a subset containing 10.7% of the original images and 78.5% of the identities. To assess the impact of this data composition, we train a standard ResNet-based network on MS1Mv3-FP with the ArcFace loss [15], without modifying either the architecture or the loss function. This setup isolates the effect of the training data composition by keeping the remaining model components fixed. The novelty of this work is therefore twofold. Methodologically, the selection criterion is conditioned on the pose distribution within each identity, rather than on class frequency, sample difficulty, or redundancy, and it is applied once, before training. Empirically, controlled comparisons against equal-size subsets, with the same backbone, loss function, and training protocol, show that the pose composition of the subset, and not only its size, contributes to the performance retained with 10.7% of the training images.

The main contributions of this work are as follows:

1. Pose-balanced sampling strategy. We formalize a sampling strategy that conditions subset construction on the per-identity head-pose distribution rather than on sample difficulty or redundancy. Applying it to MS1Mv3 yields MS1Mv3-FP, a subset of 547,202 images (10.7% of the original) covering 78.5% of the identities; we will publicly release its image list as a reusable training resource.
2. Data-efficient training. We show that an SE-ResNet100 trained from scratch on MS1Mv3-FP stays within 0.30, 0.70, and 0.86 percentage points (pp) of the same network trained on the complete MS1Mv3 and tested on CFP-FP, CPLFW, and VGG2-FP, respectively, for the best of three sampled subsets (0.40, 0.80, and 1.10 pp on average), while reducing training time from more than 100 h to approximately 11 h on the same single GPU. Equal-size control subsets built without pose balancing lose between 0.8 and 3.6 pp on average, indicating that the pose composition of the subset, and not only its size, accounts for this result.
3. Complementarity with full-dataset training. We find that fine-tuning a model pre-trained on the complete MS1Mv3 with MS1Mv3-FP yields small but consistent gains (0.07–0.27 pp) on all three cross-pose benchmarks, suggesting that the subset provides additional exposure to profile samples that remain underrepresented in the full dataset.
4. Sensitivity analysis and scope. We characterize the strategy’s sensitivity to the profile yaw-angle threshold, the backbone (depth and the use of Squeeze-and-Excitation (SE) blocks), and the two preprocessing choices (profile-side flipping and cutout). We also delimit its scope: models trained on MS1Mv3-FP remain within 0.35 pp of the reported results on frontal–frontal benchmarks, but fall further behind on age-gap verification, where the compact subset offers limited variability in factors other than pose.

The rest of this paper is organized as follows. Section 2 reviews related work on FR under pose variation and on approaches to reduce resource requirements in FR. Section 3 describes the proposed pose-balanced sampling strategy, the training protocol, and the experimental setup. Section 4 presents and discusses the results. Finally, Section 5 concludes this paper and outlines future work.

## 2. Related Work

### 2.1. FR with Pose Variation

Head pose remains one of the dominant sources of error in unconstrained FR [36]. A widely accepted explanation is that the distribution of head orientations in web-collected training sets is heavily skewed towards frontal faces, so the learned embedding is implicitly optimized for a pose range that does not match the operating conditions of cross-pose benchmarks such as CFP-FP [30] and CPLFW [29]. This discrepancy between training and evaluation conditions has also been documented in previous work [37], which reported that models achieving near-optimal performance on frontal faces degrade substantially on profile faces. Existing approaches fall into four categories: image-space normalization, feature-space transformation, pose-aware loss functions and sampling strategies, and pose-specialized architectures.

Image-space methods synthesize a frontal view before recognition, so that a single pose-agnostic model can be applied downstream. Recent formulations rely on generative adversarial networks (GANs), such as the self-supervised random mask attention GAN of Liao et al. [38]. Although visually convincing at moderate angles, these approaches are computationally expensive, and their accuracy degrades for extreme profile views, where much of the facial information is missing and the synthesis problem becomes severely ill posed [36].

Feature-space methods avoid pixel-level synthesis and instead transform the embedding itself. The Deep Residual Equivariant Mapping (DREAM) block [37] adds a pose-gated residual that maps a profile representation towards its canonical counterpart and can be attached to a ResNet backbone with negligible overhead. This idea has been extended in several directions: Mostofa et al. [39] used pose as auxiliary information through channel-wise and spatial attention blocks; Zhang et al. [40] distilled the angular knowledge of frontal features into profile features; and FCGFace [31] used frontal feature centers to guide optimization and narrow the frontal–profile gap. More recently, Stanishev et al. [32] proposed a feature-space pose frontalization module (FSPFM) driven by the estimated head pose, trained under a two-stage paradigm in which a ResNet50 backbone is first pre-trained on MS1M and then fine-tuned on paired data restricted to large yaw angles. This last work is methodologically close to ours in that the corrective stage operates on a deliberately selected, pose-conditioned subset rather than on the full training set.

A third family acts on the loss or on the sampling policy rather than on the representation. PoseFace [41] disentangles pose-invariant features using facial landmarks and adapts the angular margin as a function of the estimated yaw, assigning larger margins to profile samples precisely because they are rare. Liu et al. [42] (MvCoM) generalized this observation, showing that head pose behaves as a long-tailed factor alongside ethnicity, blur, and occlusion, and proposing a sample-level weighting scheme learned from a held-out meta set. Both works support the premise of this paper: the pose composition of the training data, and not only its volume, affects cross-pose performance.

Finally, a fourth family assigns different models to different orientation ranges. Early work by AbdAlmageed et al. [43] combined deep representations extracted from multiple pose-specific models. More recently, Perez and Perez [27] used a two-level genetic algorithm (2LGA) to design three CNN architectures specialized for small, medium, and large poses. This method reports strong results on cross-pose benchmarks, but it requires a full architecture search per pose range, with search budgets measured in hundreds of GPU-days. The present work retains the pose-aware perspective but dispenses with architecture search: a single fixed ResNet backbone is trained on a pose-balanced subset, which isolates the contribution of the data selection from that of the architecture.

### 2.2. Approaches to Reduce Resources in FR

Efforts to make FR less resource-intensive have addressed three distinct bottlenecks: the inference cost of the backbone, the cost of the classification layer during training, and the cost of the training data itself.

The first and most explored direction is architectural compression. MobileFaceNets [44] and GhostFaceNets [45] redesign the backbone around depthwise-separable convolutions and inexpensive linear operations, while PocketNet [33] automates this design through differentiable architecture search combined with multistep knowledge distillation. These models reduce the number of parameters by one or two orders of magnitude, but they consistently lose accuracy on cross-pose benchmarks, which is the regime of interest here. A different form of input-level economy was explored by Bhatta et al. [46], who showed that FR models trained on grayscale images (FR-grayscale) match their color-trained counterparts, reducing the input dimensionality by a factor of three without discarding any training samples.

The second direction targets training rather than inference. Because the memory and computation of the fully connected layer scale linearly with the number of identities, An et al. [47] proposed PartialFC, a sparsely updated variant in which only the positive class centers and a random subset of negative centers are updated at each iteration. A complementary strategy is to limit the number of parameters that are adapted at all: PETALface [48] showed that low-rank adapters modulated by image quality outperform full fine-tuning when transferring a pre-trained FR model to a new domain, while updating only 0.48% of the parameters. Both results indicate that the marginal value of updating the entire model on the entire dataset is lower than commonly assumed.

The third direction, and the closest to this work, is data-centric. Zhang and Deng [49] (CBFace) observed that indiscriminately enlarging the training set can degrade performance once the class distribution becomes long-tailed, and that a carefully selected subset may outperform the full set. DiffProb [34] formalized this idea for FR through prediction-probability-based pruning, removing up to 50% of CASIA-WebFace while maintaining, and in some settings improving, verification accuracy on LFW, CFP-FP, and IJB-C. However, existing pruning criteria are defined in terms of redundancy, label noise, or sample difficulty, and are agnostic to the geometric factor that dominates the residual error: head orientation. To the best of our knowledge, no previous study has selected a training subset explicitly conditioned on the pose distribution, nor quantified how small such a subset can be before cross-pose accuracy degrades. The present work addresses this gap.

Table 1 summarizes how the proposed strategy relates to the most closely related pose-aware and data-centric methods. Two differences emerge. First, the factor being balanced differs: data-balancing and pruning methods act across identities, or on individual samples according to their difficulty or redundancy, whereas the proposed strategy balances poses within each identity, so that every retained identity contributes both frontal and profile views. Second, the point of action differs: pose-aware methods such as PoseFace, MvCoM, and FSPFM train on the complete dataset and compensate for the pose imbalance through the loss function, the sample weights, or an additional module, whereas the proposed strategy modifies only the training data, once before training, and leaves the loss function and the architecture unchanged. The proposed strategy is therefore complementary to these methods and could, in principle, be combined with them.

## 3. Materials and Methods

### 3.1. Overview

Large-scale FR models are commonly trained on datasets containing millions of images collected in unconstrained environments. Although these datasets provide extensive identity diversity, their head-pose distribution is strongly skewed: frontal and near-frontal images vastly outnumber profile images. As a consequence, frontal–profile verification remains challenging, and simply increasing the amount of training data does not correct this skew.

The central hypothesis of this work is that learning from a compact pose-balanced dataset provides a favorable trade-off between computational efficiency and frontal–profile verification performance compared with training on the complete dataset. Figure 1 illustrates the overall framework of the proposed method, which consists of three stages: constructing the pose-balanced training subset, training a CNN on the resulting subset, and evaluating frontal–profile face verification on public benchmarks.

### 3.2. Sensor-Aware Pose-Balanced Sampling Strategy

First, we estimate the head pose of every image using the 6DRepNet algorithm [35], which predicts the yaw, pitch, and roll angles. Since the distinction between frontal and profile faces is mainly determined by horizontal head rotation, only the yaw angle is considered during sampling.

Sengupta et al. define near-profile faces as those in which the second eye is occluded, corresponding to yaw angles greater than 60^∘^, while near-frontal faces are defined as those in which both sides of the face are almost equally visible, corresponding to yaw angles within 10^∘^ [30]. Alternatively, Perez and Perez define small-pose faces as those with yaw angles below 10^∘^ and large-pose faces as those with yaw angles greater than 40^∘^ [27]. Although the 60^∘^ threshold provides a more restrictive definition of profile faces, it yields too few profile samples in large-scale FR datasets: only about 1.5% of the images in MS1Mv3 have absolute yaw angles greater than 60^∘^, according to the 6DRepNet estimates computed in this study for all 5,131,015 images. Therefore, we adopt the 40^∘^ threshold proposed in [27]. Images with absolute yaw angles between 10^∘^ and 40^∘^ are discarded: the three target benchmarks compare a near-frontal image against a profile image, so retaining intermediate poses would spend part of the image budget on a pose range that these benchmarks do not directly evaluate.

After pose classification, the dataset is balanced independently for every identity. Let $F_{i}$ and $P_{i}$ denote the sets of frontal and profile images belonging to identity *i*, respectively. The number of images retained for each pose is $N_{i}=\min(|F_{i}\left|,\right|P_{i}\left|\right)$. Subsequently, $N_{i}$ frontal images and $N_{i}$ profile images are randomly selected without replacement. Note that profile images are far scarcer than frontal ones in MS1Mv3 (Table 2); in practice, $N_{i}=\left|P_{i}\right|$ for most identities: the strategy retains essentially all available profile images and subsamples the frontal ones. The size of the resulting subset is therefore determined by the pose statistics of the source dataset rather than being a free parameter. By construction, every identity contributes an equal number of frontal and profile images to the final training set. Identities without at least one frontal and one profile image are discarded, since they cannot contribute frontal–profile pairs. Algorithm 1 summarizes the pose-balancing strategy.
**Algorithm 1** Pose-Balanced Sampling Strategy**Require:** FR dataset D, frontal threshold $\theta_{f}$ = $10^{\circ }$, profile threshold $\theta_{p}$ = $40^{\circ }$**Ensure:** Pose-balanced training subset $D_{FP}$
1:Initialize $D_{FP}\leftarrow \emptyset$2:Estimate the yaw angle of every image in D using 6DRepNet.3:**for** each identity i∈D **do**4:    $F_{i}\leftarrow \{I||yaw\left(I\right)|<\theta_{f}\}$5:    $P_{i}\leftarrow \{I||yaw\left(I\right)|>\theta_{p}\}$6:    **if** $|F_{i}|>0$ **and** $|P_{i}|>0$ **then**7:        $N_{i}\leftarrow \min(|F_{i}\left|,\right|P_{i}\left|\right)$8:        ${\hat{F}}_{i}\leftarrow \mathrm{RandomSample}\left(F_{i},N_{i}\right)$9:        ${\hat{P}}_{i}\leftarrow \mathrm{RandomSample}\left(P_{i},N_{i}\right)$10:        $D_{FP}\leftarrow D_{FP}\cup {\hat{F}}_{i}\cup {\hat{P}}_{i}$11:    **end if**12:**end for**13:**return** $D_{FP}$

### 3.3. Datasets

The experimental evaluation uses four main datasets: one dataset for training and three public benchmarks for frontal–profile face verification. The proposed pose-balanced sampling strategy is applied to the MS1Mv3 dataset to construct the training subset, while CFP-FP, CPLFW, and VGG2-FP are used exclusively for performance evaluation. Four additional benchmarks used in the generalization analysis are described in Section 4.4.

MS1Mv3 is a cleaned version of the MS-Celeb-1M dataset, containing more than five million images from approximately 85K identities [25]. Applying the proposed pose-balanced sampling strategy reduces the dataset to approximately 10.7% of the original images while preserving 78.5% of the identities. We refer to this subset as MS1Mv3-FP. Table 2 shows how MS1Mv3-FP is progressively constructed by Algorithm 1.

Figure 2 presents the distribution of frontal–profile image pairs for each identity in MS1Mv3-FP. Most identities contain fewer than five frontal–profile pairs, whereas only a small proportion have more than 20 pairs.

Performance is evaluated on the CFP-FP, CPLFW, and VGG2-FP benchmarks. CFP-FP contains 3500 positive and 3500 negative verification pairs. CPLFW extends the LFW benchmark by introducing large pose variations while preserving the demographic balance of the negative pairs; it contains 3000 positive and 3000 negative pairs. VGG2-FP consists of 2500 positive and 2500 negative frontal–profile pairs extracted from the VGGFace2 test partition. We follow the standard evaluation protocol of each benchmark.

Figure 3 compares the yaw-angle distributions of the training and evaluation datasets. The proposed MS1Mv3-FP training subset exhibits a pose distribution similar to that of CFP-FP, whereas CPLFW and VGG2-FP cover a wider range of pose variations.

### 3.4. Experimental Details

Unless otherwise stated, all experiments use SE-ResNet100, a ResNet [13] with SE blocks [50], as the backbone, given the widespread adoption of ResNet-based backbones in FR. The network is trained from scratch with the ArcFace loss using the default scale factor (s=64) and angular margin (m=0.5) [15]. Other ResNet variants trained on MS1Mv3-FP are compared in the ablation study.

Since profile images may correspond to either left or right facial views, all left-profile images are horizontally flipped so that every profile face shares the same orientation. This preprocessing is applied consistently during both training and evaluation. All face images are resized to 112×112 pixels, and the network produces a 512-dimensional embedding for each input image.

Motivated by the observation in ARFace [51] that not every facial region contributes positively to unconstrained FR, we apply cutout data augmentation [52] during training, using four square masks of 28×28 pixels, to improve the robustness of the learned representations.

The network is trained for 30 epochs using the Adam optimizer with an initial learning rate of 0.1, which is reduced to 0.01 and 0.001 at epochs 15 and 25, respectively. All experiments are implemented in PyTorch (version 2.1.0) and run on a single NVIDIA RTX 4090 GPU with a batch size of 128.

## 4. Results and Discussion

### 4.1. Contextual Comparison with Published Results

Table 3 compares the proposed method, using ResNet100 and SE-ResNet100 backbones, with 19 representative FR approaches on CFP-FP, CPLFW, and VGG2-FP. The compared methods are grouped into three categories according to their main objective: general FR, pose-robust FR, and resource-efficient FR. Since these methods differ in training data, backbone, and loss function, this comparison does not isolate the effect of the sampling strategy. The controlled comparisons in Section 4.2, in which only the training data change, constitute the primary evidence of this study.

To the best of our knowledge, only DiffProb [34], CBFace [49], and the proposed method are trained on a subset of their source dataset. Specifically, the proposed approach uses only 10.7% of the original MS1Mv3 training images, all of which are frontal or profile faces. In comparison, the DiffProb configuration reported for CFP-FP and CPLFW retains approximately 75% of CASIA-WebFace (about 370K images), while CBFace is trained on a class-balanced subset containing approximately 500K images from the MillionCeleb dataset. With a comparable number of images, our method outperforms DiffProb and CBFace by 3.26 and 2.49 pp, respectively, on CFP-FP, and by 2.90 and 2.53 pp on CPLFW. These results suggest that balancing the number of frontal and profile images per identity is more beneficial for large-pose FR than reducing the training set size or balancing class size alone, although the compared methods also differ in their backbone and source dataset. Section 4.2 isolates the effect of the selection strategy under a fixed backbone.

Among the general FR methods, ArcFace is the most relevant comparison, since our method uses the same loss function. With the same ResNet100 backbone, the proposed method is 1.31 and 1.86 pp below ArcFace on CFP-FP and CPLFW, respectively. However, with SE-ResNet100, the gap on CFP-FP narrows to only 0.06 pp, and the proposed method slightly exceeds ArcFace on CPLFW (+0.02 pp). Compared with the two most recent general FR methods, TopoFR and AdaFace+IIC, both trained on the complete MS1Mv2 dataset with ResNet100, the proposed SE-ResNet100 model achieves comparable accuracy on CFP-FP (98.73% vs. 98.71% and 98.41%, respectively), whereas it remains 0.56 and 0.25 pp below them on CPLFW, and 1.06 pp below AdaFace+IIC on VGG2-FP.

Among the pose-robust FR methods, 2LGA achieves the highest verification accuracy on all three benchmarks. Nevertheless, the proposed method remains competitive, trailing by only 0.37, 1.22, and 0.86 pp on CFP-FP, CPLFW, and VGG2-FP, respectively. Note, however, that 2LGA employs a neural architecture search framework to discover three specialized architectures for small-, medium-, and large-pose FR, whereas the proposed method uses a conventional backbone without architectural modifications or pose-specific feature generation. Compared with the remaining pose-robust methods, the proposed approach achieves the highest accuracy on CFP-FP and VGG2-FP and is only 0.60 pp below FSPFM on CPLFW. Moreover, FSPFM requires an additional feature-frontalization module, whereas our method compares the embeddings directly, without any additional module.

Within the resource-efficient FR category, the proposed method outperforms the published results of all competing approaches except FR-grayscale, which is trained on the complete WebFace4M dataset and reduces computational cost by using grayscale images rather than reducing the amount of training data. In contrast, the proposed approach achieves comparable verification performance while reducing the training data by approximately 90%, indicating a favorable trade-off between recognition accuracy and training efficiency.

Overall, although the proposed method does not achieve the highest verification accuracy among all state-of-the-art approaches, it consistently provides competitive performance while requiring only 10.7% of the original MS1Mv3 training images. A balanced frontal–profile subset thus appears to retain much of the pose information relevant to large-pose face verification.

The comparison in Table 3 involves methods trained on different datasets and with different backbones. To isolate the effect of the sampling strategy, we now compare training regimes under a fixed backbone and a fixed loss function.

### 4.2. Controlled Comparison: Effect of Training Data Selection

#### 4.2.1. MS1Mv3 vs. MS1Mv3-FP

Table 4 compares the performance of SE-ResNet100 under three training settings: the balanced frontal–profile subset (MS1Mv3-FP), the complete MS1Mv3 dataset, and the complete MS1Mv3 dataset followed by fine-tuning with MS1Mv3-FP. Training exclusively with MS1Mv3-FP reduces verification accuracy by 0.30, 0.70, and 0.86 pp on CFP-FP, CPLFW, and VGG2-FP, respectively, for the best sampled subset (MS1Mv3-FP1), and by 0.40, 0.80, and 1.10 pp on average over the three sampled subsets, compared with training on the complete MS1Mv3 dataset. However, this small loss in accuracy comes with a substantial reduction in training time. Specifically, training with MS1Mv3-FP takes approximately 11 h, whereas training with the full MS1Mv3 dataset takes more than 100 h. Estimating the head pose of every MS1Mv3 image with 6DRepNet takes approximately 10 h, a one-time preprocessing cost. Inference on the test sets takes approximately 10 s; it depends only on the model architecture and is therefore independent of the training subset.

Additionally, fine-tuning the model initially trained on the complete MS1Mv3 dataset with MS1Mv3-FP improves performance by 0.07, 0.27, and 0.14 pp on CFP-FP, CPLFW, and VGG2-FP, respectively.

To assess the variability associated with the sampling procedure, we generated three versions of MS1Mv3-FP using different random seeds and trained the corresponding models under the same configuration. Across these three subsets, the mean verification accuracies were 98.63±0.13% on CFP-FP, 93.13±0.17% on CPLFW, and 94.50±0.34% on VGG2-FP, where ± denotes the sample standard deviation across the three subsets.

These results point to two benefits of the proposed frontal–profile balancing strategy for large-pose FR. First, it yields a compact training subset that approaches the performance of the complete dataset at a fraction of the training time. Second, the fine-tuning results indicate that MS1Mv3-FP still provides useful information for large-pose face verification after the model has been trained on the complete MS1Mv3 dataset. A plausible explanation is that the balanced distribution of frontal and profile images provides greater exposure to profile information that is underrepresented in the original MS1Mv3 dataset.

#### 4.2.2. Equal-Size Subset Comparison

To investigate whether the performance of MS1Mv3-FP can be attributed solely to the use of a smaller training set, five equal-size control subsets were constructed, each containing 547,202 images. Two subsets were randomly sampled from the pool of frontal and profile images (F+P), and a third was sampled from identities containing at least one frontal and one profile image, without explicitly balancing the two poses. The remaining two subsets were randomly sampled from the complete MS1Mv3 dataset without pose-based filtering. Table 5 reports the performance of SE-ResNet100 after training on these control subsets, together with the mean performance of MS1Mv3-FP over its three sampled subsets, so that the comparison does not rely on a single sampling of the proposed subset.

The three controls sampled from F+P achieve lower accuracy than MS1Mv3-FP across all three benchmarks. The two controls sampled from the complete MS1Mv3 dataset perform better than the F+P controls but still fall short of MS1Mv3-FP by 1.19, 1.46, and 0.27 pp on average for CFP-FP, CPLFW, and VGG2-FP, respectively. Even the best control (Control-5) remains 1.05 and 1.03 pp below MS1Mv3-FP on CFP-FP and CPLFW, respectively, whereas the 0.20 pp difference on VGG2-FP is within the variability observed across the sampled subsets of MS1Mv3-FP. Subset size alone therefore does not explain the observed performance differences. However, the control subsets also differ in identity composition and image distribution, so these comparisons do not fully isolate the effect of pose balancing.

The proposed strategy fixes the frontal-to-profile ratio at 1:1, and other sampling ratios were not evaluated. This choice follows from the scarcity of profile images: since $N_{i}=\left|P_{i}\right|$ for most identities, MS1Mv3-FP already retains essentially all available profile images. Any ratio other than 1:1 would therefore either add frontal images, which increases the subset size and confounds the effect of the ratio with that of the size, or remove profile images at a fixed size, which discards the scarcest samples. Nevertheless, the controls sampled at random from F+P (Control-1 and Control-2) provide an equal-size reference at the natural pose ratio of this pool. Since the pool contains 2,585,593 frontal and 279,837 profile images, the expected frontal-to-profile ratio of these controls is approximately 9:1. Their lower accuracy is therefore consistent with a benefit of moving from the natural ratio to a balanced one at a fixed subset size.

### 4.3. Ablation Study

#### 4.3.1. Profile Threshold Sensitivity Analysis

To evaluate the sensitivity of the proposed sampling strategy to the profile yaw-angle threshold, we conducted additional experiments using $\theta_{p}\in \left\{30^{\circ },40^{\circ },50^{\circ },60^{\circ }\right\}$, while keeping the frontal threshold fixed at $\theta_{f}=10^{\circ }$. Table 6 reports the number of selected images and identities, together with the verification accuracy on CFP-FP, CPLFW, and VGG2-FP. With $\theta_{p}=30^{\circ }$, the selected subset contains 1,147,502 images from 79,814 identities, yielding the highest accuracy on all three benchmarks among the evaluated settings. Increasing the threshold to $40^{\circ }$ reduces the subset to 547,202 images from 67,504 identities, while retaining most of the verification accuracy. This configuration, adopted in the proposed method, represents a trade-off between training set size and verification accuracy.

When the threshold increases to $50^{\circ }$ and $60^{\circ }$, the selected subsets become substantially smaller, containing 283,838 and 153,514 images, respectively. Verification accuracy decreases accordingly on all three benchmarks, with a sharper decline at $60^{\circ }$. These results indicate that the profile threshold affects both the composition and the size of the training subset, and consequently verification performance. In particular, a more restrictive profile criterion reduces the number of available training samples and may limit the representation of large-pose variations.

#### 4.3.2. Backbone Architectures

Table 7 compares the performance of five ResNet-based backbones trained with MS1Mv3-FP. Adding SE blocks consistently improves performance over the corresponding plain ResNet. For ResNet50, the SE block improves performance by 1.41, 1.65, and 0.52 pp on CFP-FP, CPLFW, and VGG2-FP, respectively. Similarly, SE-ResNet100 improves over ResNet100 by 1.25, 1.88, and 1.08 pp on the three benchmarks. SE blocks therefore appear to benefit large-pose face verification. Finally, SE-ResNet152 performs slightly worse than SE-ResNet100 across all three benchmarks, with reductions of 0.26, 0.16, and 0.36 pp on CFP-FP, CPLFW, and VGG2-FP, respectively. This supports the choice of SE-ResNet100 for the main experiments, since the deeper SE-ResNet152 offers no advantage.

#### 4.3.3. Effect of Cutout and Flipping

Table 8 evaluates the contribution of two image-processing strategies used in the proposed training pipeline: horizontally flipping left-profile images during preprocessing so that all profiles share the same orientation, and applying cutout during training. The best performance is obtained when both strategies are applied, achieving 98.73%, 93.23%, and 94.74% on CFP-FP, CPLFW, and VGG2-FP, respectively. When the left-profile images are not flipped during preprocessing, performance decreases by an average of 0.43 pp across the three benchmarks, while removing cutout during training results in an average reduction of 0.80 pp. Both strategies therefore contribute to the performance of the proposed training pipeline.

### 4.4. Generalization to Other Benchmarks

Although this work focuses on large-pose face verification, we further evaluate the best-performing model on other commonly used face verification benchmarks to assess the generalization of MS1Mv3-FP beyond large-pose scenarios. Specifically, we consider frontal–frontal verification on CFP-FF and LFW, as well as age-gap verification on CALFW and AgeDB-30. The results are reported in Table 9 and are compared with representative state-of-the-art methods.

On frontal–frontal verification, the SE-ResNet100 trained exclusively with MS1Mv3-FP achieves 99.60% on CFP-FF and 99.45% on LFW. These results are competitive with those reported by state-of-the-art methods, with differences of at most 0.23 pp on CFP-FF and 0.35 pp on LFW. Thus, although designed for large-pose verification, the proposed strategy does not substantially compromise frontal face verification.

On age-gap benchmarks, the SE-ResNet100 achieves 94.87% on CALFW and 94.42% on AgeDB-30, compared with 95.63% and 97.17% reported by PocketNet, respectively. One possible explanation is the smaller number of images per identity in MS1Mv3-FP: 8.1 images per identity on average, compared with 59.7 in MS1Mv3 (Table 2), with most identities contributing fewer than five frontal–profile pairs (Figure 2). Fewer images per identity reduce the likelihood of observing the same person at different ages, which age-gap benchmarks require. Since MS1Mv3 does not provide age annotations and PocketNet also differs in backbone and training data, this explanation remains a hypothesis.

### 4.5. Failure-Case Analysis

To identify where models trained on the proposed subset fail, we compared, pair by pair, the predictions of the SE-ResNet100 trained on MS1Mv3-FP with those of the same network trained on the complete MS1Mv3 dataset. Figure 4 reports the error rate of both models as a function of the absolute yaw difference between the two images of each pair, estimated with 6DRepNet. Table 10 summarizes the composition of the errors, the errors shared by both models, and the exact McNemar test [53], which compares the two models on the pairs that only one of them misclassifies.

Three observations emerge. First, most failures are shared: 67% and 65% of the errors of the MS1Mv3-FP model on CPLFW and VGG2-FP, respectively, as well as 48% on CFP-FP, are also made by the model trained on the complete dataset, which indicates that they correspond to intrinsically difficult pairs. Second, the additional errors of MS1Mv3-FP are mainly false positives, that is, impostor pairs accepted as the same identity (60 vs. 31 on CPLFW and 52 vs. 13 on VGG2-FP), whereas false negatives increase only slightly. This pattern is consistent with the lower identity diversity of MS1Mv3-FP, which retains 78.5% of the identities with 8.1 images per identity on average (Table 2), and suggests that the subset mainly weakens the separation between different identities rather than the matching of frontal and profile views of the same identity. Third, although the model trained on the complete dataset makes significantly fewer errors overall (*p* < 0.01 on all three benchmarks), the gap does not increase with the pose difference: for yaw differences of $60^{\circ }$ or more, the differences between the two models are not statistically significant.

This analysis also exposes a failure mode of the pose estimator. Since CFP-FP contains only frontal–profile pairs, the 20 pairs with a yaw difference below $20^{\circ }$ indicate pose-estimation errors of 6DRepNet; both models misclassify three of them. Because the proposed strategy relies on the same estimator to select the training images, such errors may also affect the composition of MS1Mv3-FP.

Figure 5 shows randomly selected failure cases, that is, pairs misclassified by the MS1Mv3-FP model and correctly classified by the model trained on the complete dataset. Among the false positives (Figure 5a), the confused identities in CPLFW, whose impostor pairs are selected to share demographic attributes, have a similar appearance, whereas in CFP-FP and VGG2-FP, some accepted pairs differ markedly in age or ethnicity when one of the images is a profile. Among the false negatives (Figure 5b), most pairs combine the pose difference with intra-class variations, such as sunglasses, headwear, large age gaps, and low resolution. These variations are less represented in MS1Mv3-FP, which contains 8.1 images per identity on average, compared with 59.7 in MS1Mv3 (Table 2). This is consistent with the lower performance observed on age-gap benchmarks (Section 4.4).

These results delimit the claims of this study. Training on MS1Mv3-FP does not introduce a specific weakness at large pose differences, but the reduced identity diversity increases the false acceptance of different identities. Moreover, the benchmarks do not provide annotations for occlusion, resolution, or illumination; therefore, the effect of these factors was only examined qualitatively and remains a limitation of this study.

## 5. Conclusions

This paper shows that a compact, pose-balanced training subset can achieve competitive large-pose face verification performance while substantially reducing training time. We constructed MS1Mv3-FP by selecting frontal and profile images from MS1Mv3 and balancing their representation within each identity. Experiments with five backbones showed that this subset, which contains approximately 10.7% of the original MS1Mv3 images, retains most of the large-pose verification accuracy of the complete dataset. In a controlled comparison against equal-size subsets (547,202 images), accuracy dropped by 0.8–3.6 pp on average when pose balance was not enforced, indicating that the pose composition, and not only the subset size, contributes to this result. By improving the composition of the training data while reducing training requirements, the proposed strategy offers a data-efficient approach to developing face verification models for camera-based sensing systems, where computational resources are often limited.

Although the proposed approach does not achieve the highest accuracy among all compared methods, it offers a favorable trade-off between recognition performance and training efficiency. Its accuracy is comparable to the published results of resource-efficient and pose-robust FR methods while using only 10.7% of the training images, but these comparisons involve different training settings. Under controlled conditions, replacing MS1Mv3 with MS1Mv3-FP causes only a moderate decrease in verification accuracy, whereas fine-tuning a model pre-trained on the complete MS1Mv3 with MS1Mv3-FP improves accuracy on all evaluated large-pose benchmarks. Balancing frontal and profile images therefore appears to emphasize pose information that is underrepresented in the original training distribution.

Finally, although MS1Mv3-FP was designed for large-pose face verification, models trained on it also remain competitive on frontal–frontal benchmarks, indicating that pose balancing does not impair the learning of discriminative representations for frontal faces. Performance on age-gap benchmarks is comparatively lower, which we hypothesize is related to the smaller number of images per identity in MS1Mv3-FP (8.1 vs. 59.7 in MS1Mv3). Overall, pose balancing provides an effective alternative to training on the complete dataset for large-pose face verification, while retaining competitive performance on frontal–frontal verification.

As future work, the proposed sampling strategy could be evaluated on larger datasets, such as WebFace4M and WebFace12M, to determine whether the benefits of pose balancing generalize to greater image and identity diversity. Another direction is the generation of synthetic profile images: instead of subsampling frontal images to match the available profile samples, synthetic profiles could augment the profile subset and thus preserve more of the original training data. The effect of intermediate frontal-to-profile ratios, which would require either enlarging the subset or discarding profile images, also remains to be evaluated. Finally, a direct analysis of the age diversity of MS1Mv3-FP, for example, with an age estimator, would help clarify the lower performance on age-gap benchmarks.

## Figures and Tables

**Figure 1:**
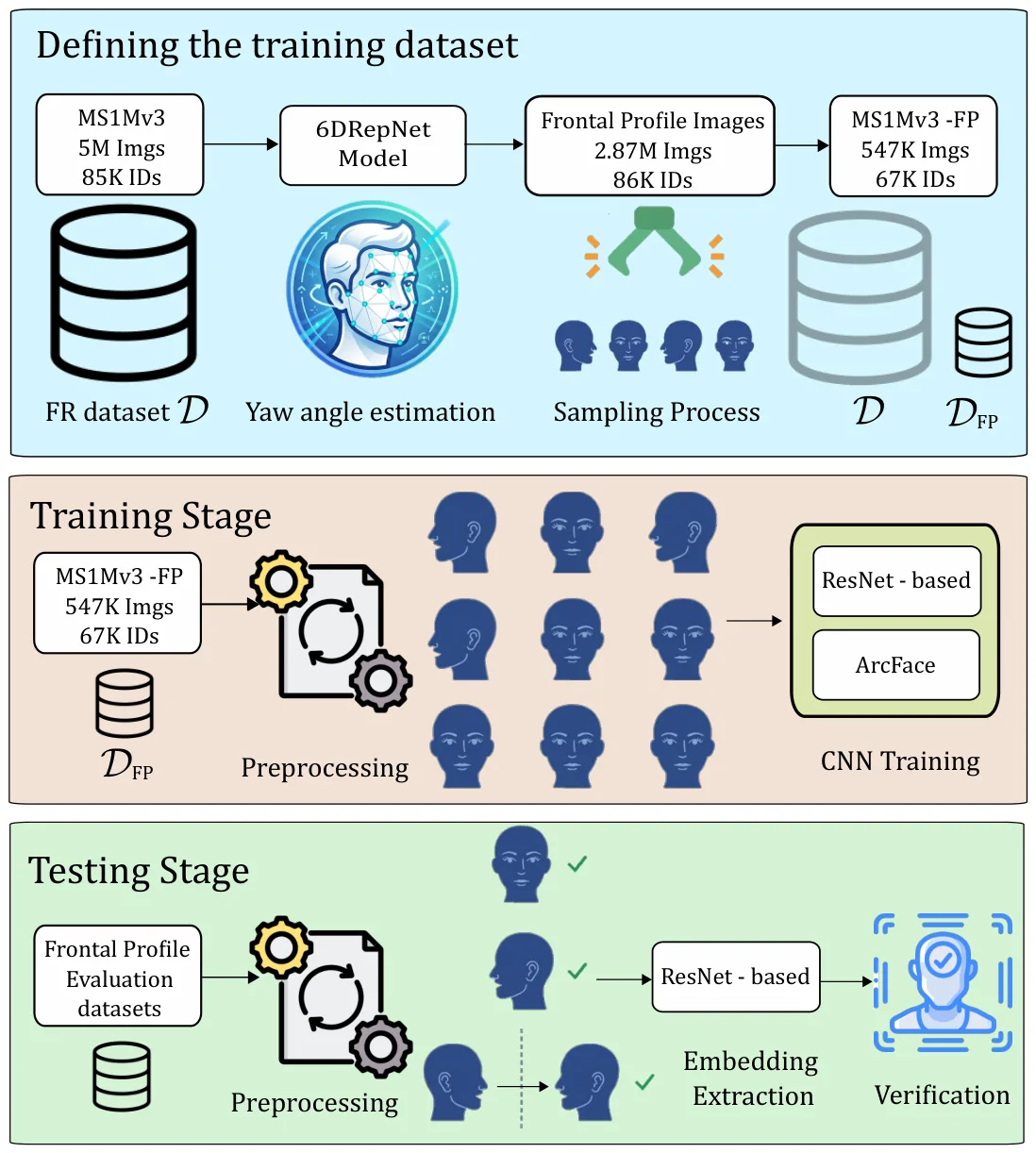
General framework of the proposed method. The first stage constructs the pose-balanced frontal–profile subset; the second trains a ResNet-based architecture on it; the third evaluates the trained model on face verification benchmarks with pose variation.

**Figure 2:**
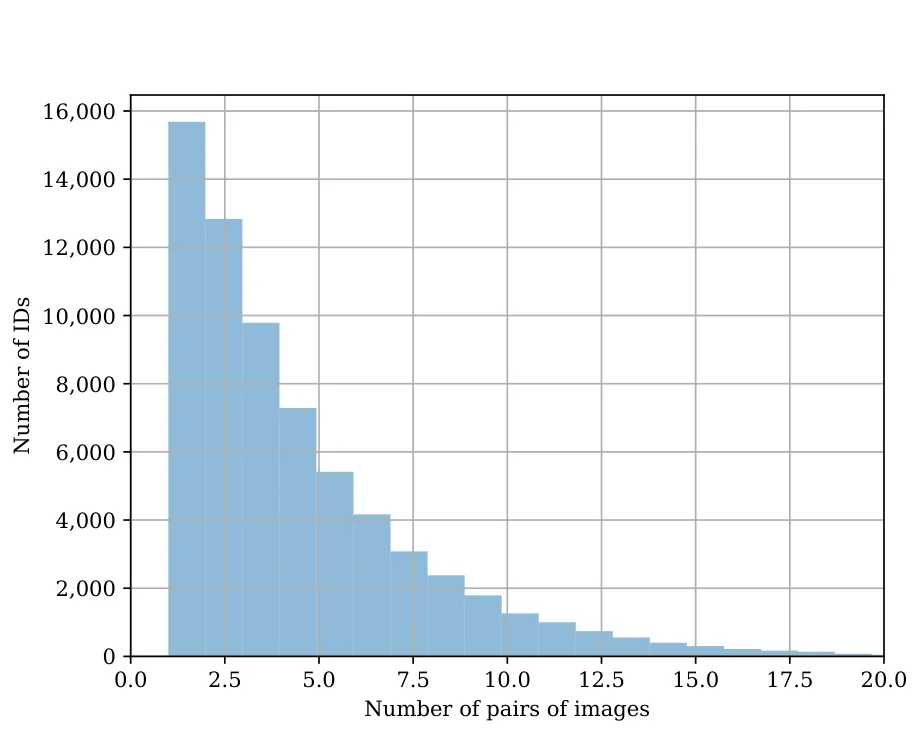
Distribution of the number of frontal–profile image pairs per identity in MS1Mv3-FP. The horizontal axis is truncated at 20 pairs; the maximum is 60.

**Figure 3:**
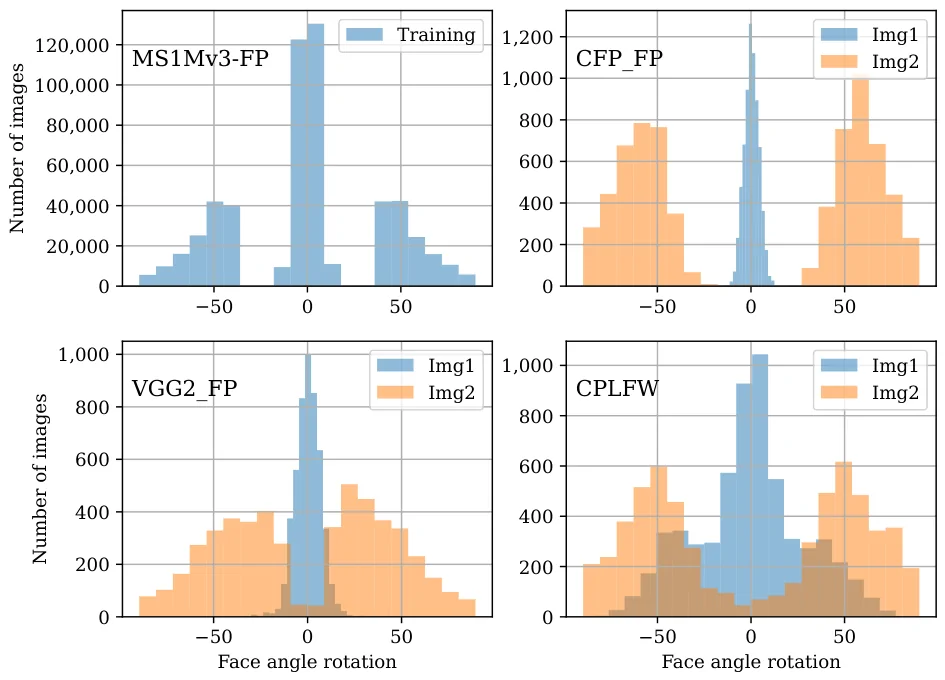
Yaw-angle distributions of the training and evaluation datasets: MS1Mv3-FP (training) and CFP-FP, VGG2-FP, and CPLFW (testing). For the evaluation sets, Img1 and Img2 denote the first and second images of each verification pair.

**Figure 4:**
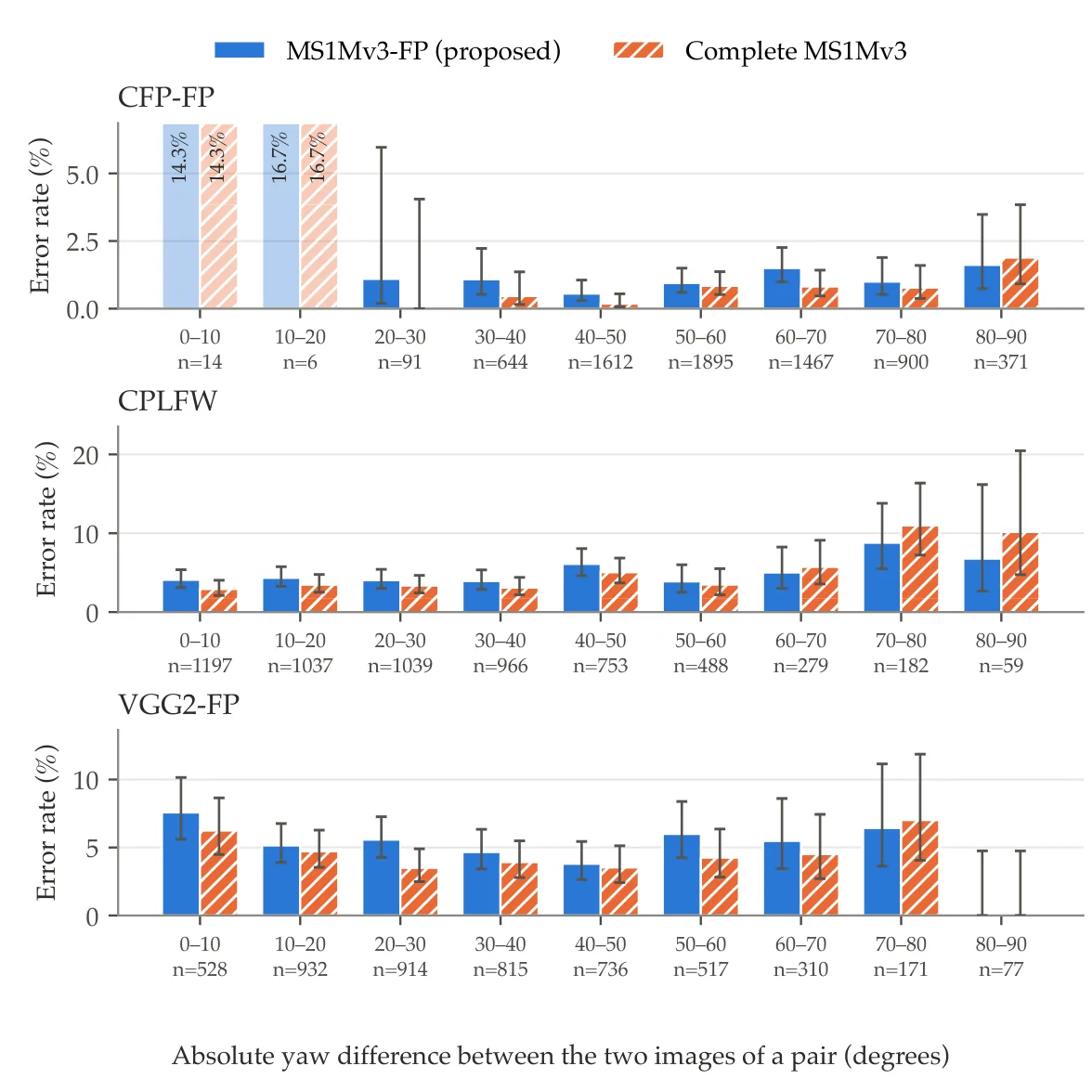
Verification error rate of SE-ResNet100 trained on MS1Mv3-FP and on the complete MS1Mv3 dataset as a function of the absolute yaw difference between the two images of a pair. Whiskers show 95% Wilson confidence intervals, and n is the number of pairs per bin. Faded bars indicate bins with fewer than 30 pairs, whose values are printed when they exceed the axis.

**Figure 5:**
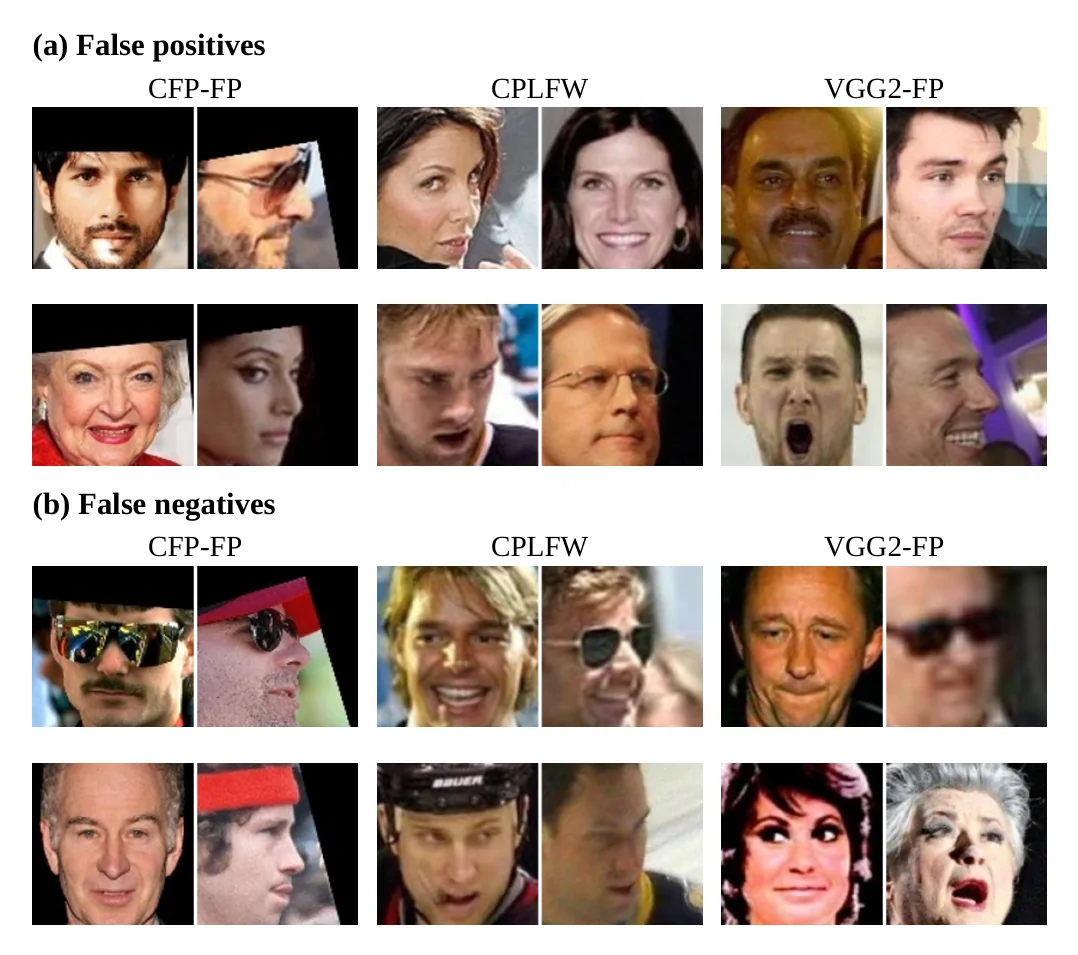
Randomly selected failure cases of SE-ResNet100 trained on MS1Mv3-FP that are correctly classified by the model trained on the complete MS1Mv3 dataset: (**a**) false positives (impostor pairs accepted) and (**b**) false negatives (genuine pairs rejected). Left profiles are horizontally flipped, and the black regions result from the alignment provided with the benchmarks.

**Table 1:** Positioning of the proposed strategy with respect to the most closely related pose-aware and data-centric methods.

Method	Balanced or Selected Factor	Criterion	Acts On	Training Data
PoseFace [41]	Pose	Estimated yaw and facial landmarks	Angular margin of the loss	Complete dataset
MvCoM [42]	Pose, ethnicity, blur, and occlusion	Weights learned from a held-out meta set	Per-sample loss weights	Complete dataset
FSPFM [32]	Pose	Estimated head pose	Feature-space module fine-tuned on large-yaw pairs	Complete dataset, then a pose-restricted subset
CBFace [49]	Identity (class size)	Class distribution	Composition of the training set	Selected subset
DiffProb [34]	Sample redundancy and difficulty	Prediction probability	Composition of the training set	Up to 50% of the data removed
Ours	Pose within each identity	Estimated yaw (frontal vs. profile)	Composition of the training set	10.7% of MS1Mv3

**Table 2:** Progressive construction of the MS1Mv3-FP subset. Percentages are relative to the complete MS1Mv3 dataset.

Subset	Images		Identities		Images per Identity		
	N	%	N	%	Min	Max	Avg
MS1Mv3	5,131,015	100.0	85,949	100.0	11	318	59.7
Profile set	279,837	5.5	67,611	78.6	1	67	4.1
Frontal set	2,585,593	50.4	85,688	99.7	1	176	30.2
MS1Mv3-FP	547,202	10.7	67,504	78.5	2	120	8.1

**Table 3:** Comparison with state-of-the-art methods on CFP-FP, CPLFW, and VGG2-FP. Methods are grouped into three categories: general FR, pose-robust FR, and resource-efficient FR approaches. The best and second-best results in each category are highlighted in **bold** and underlined, respectively. For the proposed method, the best of the three sampled subsets is reported; the mean is given in Table 4. Results of the compared methods are taken from their original publications; since training data, backbones, and loss functions differ, these results are not directly comparable and are reported for reference only.

Method	Backbone	Training	CFP-FP	CPLFW	VGG2-FP
ArcFace [15]	ResNet100	MS1Mv3 (5.1M)	98.79	93.21	-
CurricularFace [16]	ResNet100	MS1Mv2 (5.8M)	98.37	93.13	-
AdaFace [17]	ResNet100	MS1Mv3 (5.1M)	99.03	**93.93**	-
UnifiedFace [18]	ResNet100	MS1Mv3 (5.1M)	97.77	92.65	95.36
ARFace [51]	ResNet50	VGGFace2 (3.3M)	95.52	89.2	-
PartialFC [47]	ResNet100	WebFace4M (4M)	**99.06**	-	-
TopoFR [19]	ResNet100	MS1Mv2 (5.8M)	98.71	93.79	-
AdaFace + IIC [20]	ResNet100	MS1Mv2 (5.8M)	98.41	93.48	**95.80**
FCGFace [31]	ResNet50	MS1M-IBUG (3.8M)	96.34	90.07	93.72
FSPFM [32]	ResNet50	MS1Mv3 (5.1M)	98.67	93.83	-
PoseFace [41]	ResNet34	MS1Mv2 (5.8M)	94.84	88.65	-
MvCoM [42]	ResNet100	MS1Mv2 (5.8M)	98.37	92.75	-
2LGA [27]	NAS-based	MS1Mv2 (5.8M)	**99.1**	**94.45**	**95.6**
PocketNet [33]	NAS-based	MS1Mv2 (5.8M)	95.66	90.03	-
DiffProb [34]	ResNet50	CASIA-WebFace (0.37M)	95.47	90.33	-
PETALface [48]	Swin-B	WebFace4M (4M)	96.37	93.18	-
CBFace [49]	ResNet18	BUPT-CBFace (0.5M)	96.24	90.70	-
FR-grayscale [46]	ResNet100	WebFace4M (4M)	**99.17**	**94.52**	-
GhostFaceNetv2 [45]	MS1Mv3 (5.1M)		94.22	90.1	**92.76**
Ours	ResNet100	MS1Mv3-FP (0.54M)	97.48	91.35	93.66
Ours	SE-ResNet100	MS1Mv3-FP (0.54M)	98.73	93.23	94.74

**Table 4:** Comparison of SE-ResNet100 trained on MS1Mv3-FP, complete MS1Mv3, and MS1Mv3 followed by fine-tuning with MS1Mv3-FP. All models share the same backbone, loss function, and training protocol; only the training data differ.

Datasets	CFP-FP	CPLFW	VGG2-FP
MS1Mv3-FP1	98.73	93.23	94.74
MS1Mv3-FP2	98.68	93.23	94.66
MS1Mv3-FP3	98.49	92.93	94.11
Mean ± SD	98.63 ± 0.13	93.13 ± 0.17	94.50 ± 0.34
MS1Mv3	99.03	93.93	95.60
MS1Mv3 + MS1Mv3-FP1 fine-tuning	99.10	94.20	95.74
MS1Mv3 + MS1Mv3-FP2 fine-tuning	99.04	93.83	95.60
MS1Mv3 + MS1Mv3-FP3 fine-tuning	99.06	94.13	95.52
Mean ± SD	99.07 ± 0.03	94.05 ± 0.20	95.62 ± 0.11

**Table 5:** Comparison of results on CFP-FP, CPLFW, and VGG2-FP using SE-ResNet100 trained on equal-size subsets containing 547,202 images. MS1Mv3-FP is reported as the mean ± sample standard deviation over the three sampled subsets of Table 4. All models share the same backbone, loss function, and training protocol; only the training data differ.

Datasets	Selection Strategy	CFP-FP	CPLFW	VGG2-FP
MS1Mv3-FP (mean)	Pose-balanced (proposed)	98.63 ± 0.13	93.13 ± 0.17	94.50 ± 0.34
Control-1	Random from F+P	95.27	88.61	92.28
Control-2	Random from F+P	95.17	88.16	92.24
Control-3	Identity-filtered, unbalanced	96.38	90.02	93.62
Control-4	Random from complete MS1Mv3	97.31	91.25	94.16
Control-5	Random from complete MS1Mv3	97.58	92.10	94.30

**Table 6:** Effect of the profile yaw-angle threshold, with the frontal threshold fixed at $\theta_{f}=10^{\circ }$.

$\theta_{p}$ Threshold	Images	Identities	CFP-FP	CPLFW	VGG2-FP
$30^{\circ }$	1,147,502	79,814	98.77	94.11	95.46
$40^{\circ }$	547,202	67,504	98.73	93.23	94.74
$50^{\circ }$	283,838	51,637	96.74	90.23	92.68
$60^{\circ }$	153,514	36,803	90.27	83.50	87.10

**Table 7:** Comparison of results on CFP-FP, CPLFW, and VGG2-FP after training different backbones with MS1Mv3-FP.

Backbone	CFP-FP	CPLFW	VGG2-FP
ResNet50	96.91	90.60	93.80
ResNet100	97.48	91.35	93.66
SE-ResNet50	98.32	92.25	94.32
SE-ResNet100	98.73	93.23	94.74
SE-ResNet152	98.47	93.07	94.38

**Table 8:** Effect of profile flipping and cutout on CFP-FP, CPLFW, and VGG2-FP.

Training Configuration	CFP-FP	CPLFW	VGG2-FP
w/o cutout	98.04	92.30	93.96
w/o flip	98.29	92.70	94.42
w/cutout and w/flip	98.73	93.23	94.74

**Table 9:** Results on other verification benchmarks for models trained on MS1Mv3-FP, compared with state-of-the-art methods.

Method	CFP-FF	LFW	CALFW	AgeDB-30
UnifiedFace	99.81	99.8	96.17	-
PocketNet	99.58	-	95.63	97.17
ArcFace	99.81	-	95.45	-
AdaFace	99.83	-	96.02	-
Ours (ResNet100)	99.11	99.05	92.95	90.82
Ours (SE-ResNet100)	99.6	99.45	94.87	94.42

**Table 10:** Error composition of SE-ResNet100 trained on MS1Mv3-FP and on the complete MS1Mv3 dataset (MS1Mv3-FP/complete MS1Mv3). Shared errors are pairs misclassified by both models, and exclusive errors are pairs misclassified by only one of them. The *p*-values correspond to the exact McNemar test on the exclusive errors, computed over all pairs and over the pairs with an absolute yaw difference of at least $60^{\circ }$.

				All Pairs		Yaw Difference $\geq 60^{\circ }$	
**Benchmark**	**False Positives**	**False Negatives**	**Shared**	**Exclusive**	***p*** **-Value**	**Exclusive**	***p*** **-Value**
CFP-FP	15/9	60/42	36	39/15	0.001	22/11	0.080
CPLFW	60/31	213/202	184	89/49	<0.001	5/13	0.096
VGG2-FP	52/13	212/202	171	93/44	<0.001	7/5	0.774

## Data Availability

This study analyzed the MS1Mv3 training dataset and the CFP-FP, CPLFW, VGG2-FP, CFP-FF, LFW, CALFW, and AgeDB-30 benchmarks, all of which are available from their original sources. The MS1Mv3-FP is a derived subset constructed from MS1Mv3 in this study. The list of images that compose MS1Mv3-FP, together with their estimated yaw angles obtained with 6DRepNet, will be made publicly available upon publication. The original images must be obtained from the MS1Mv3 distribution under its license terms.

## Abbreviations

The following abbreviations are used in this manuscript:

2LGA	Two-Level Genetic Algorithm
AgeDB-30	Age Database, 30-year protocol
CALFW	Cross-Age Labeled Faces in the Wild
CFP-FF	Celebrities in Frontal-Profile, Frontal–Frontal Protocol
CFP-FP	Celebrities in Frontal-Profile, Frontal–Profile Protocol
CPLFW	Cross-Pose Labeled Faces in the Wild
FP	Frontal–Profile
FR	Face Recognition
FSPFM	Feature-Space Pose Frontalization Module
GAN	Generative Adversarial Network
IIC	Intra-Class Incoherence Constraint
LFW	Labeled Faces in the Wild
MS1M	MS-Celeb-1M
MS1Mv3-FP	Pose-balanced Frontal–Profile Subset of MS1Mv3
pp	Percentage Points
SE	Squeeze-and-Excitation
VGG2-FP	VGGFace2 frontal–profile protocol
